# Highly Reversible Zn Metal Anodes Enabled by Increased Nucleation Overpotential

**DOI:** 10.1007/s40820-023-01136-z

**Published:** 2023-07-06

**Authors:** Zhengqiang Hu, Fengling Zhang, Anbin Zhou, Xin Hu, Qiaoyi Yan, Yuhao Liu, Faiza Arshad, Zhujie Li, Renjie Chen, Feng Wu, Li Li

**Affiliations:** 1 Beijing Key Laboratory of Environmental Science and Engineering, School of Materials Science & Engineering, Beijing Institute of Technology, Beijing, 100081 People’s Republic of China; 2 Collaborative Innovation Center of Electric Vehicles in Beijing, Beijing, 100081 People’s Republic of China; 3 Advanced Technology Research Institute, Beijing Institute of Technology, Jinan, 250300 People’s Republic of China

**Keywords:** Nucleation overpotential, Complexing agent, Zn batteries, Zn deposition

## Abstract

Dendrite formation severely compromises further development of zinc ion batteries. Increasing the nucleation overpotential plays a crucial role in achieving uniform deposition of metal ions. However, this strategy has not yet attracted enough attention from researchers to our knowledge. Here, we propose that thermodynamic nucleation overpotential of Zn deposition can be boosted through complexing agent and select sodium L-tartrate (Na-L) as example. Theoretical and experimental characterization reveals L-tartrate anion can partially replace H_2_O in the solvation sheath of Zn^2+^, increasing de-solvation energy. Concurrently, the Na^+^ could absorb on the surface of Zn anode preferentially to inhibit the deposition of Zn^2+^ aggregation. In consequence, the overpotential of Zn deposition could increase from 32.2 to 45.1 mV with the help of Na-L. The Zn-Zn cell could achieve a Zn utilization rate of 80% at areal capacity of 20 mAh cm^−2^. Zn-LiMn_2_O_4_ full cell with Na-L additive delivers improved stability than that with blank electrolyte. This study also provides insight into the regulation of nucleation overpotential to achieve homogeneous Zn deposition.

**Supplementary Information:**

The online version contains supplementary material available at 10.1007/s40820-023-01136-z.

## Highlights

- The nucleation overpotential was regulated by sodium L-tartrate to drive smaller critical size of Zn nucleus and accelerate the nucleation rate.
- The L-tartrate anions and sodium ions can increase de-solvation energy barrier suitably and hinder the agglomerative Zn deposition resepectively.
- Nucleation overpotential in modified electrolyte could increase from 28.3 to 45.9 mV, and high Zn utilization rate of 80% at current density of 10 mA cm^−2^ can be achieved.

**Supplementary Information:**

The online version contains supplementary material available at 10.1007/s40820-023-01136-z.

## Graphical abstract

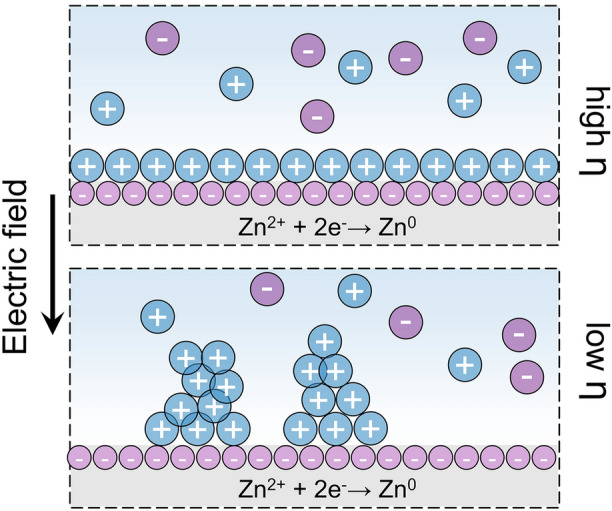

## Introduction

Rechargeable aqueous Zn ion batteries (ZIBs) are the most attractive candidate for next-generation energy storage technology owing to its high abundance, inherent safety, and environmental friendliness [1–7]. Additionally, Zn anode particularly features large theoretical capacity (820 mAh g^−1^, 5855 mAh cm^−3^), low toxicity, and moderate redox potential (− 0.76 V vs. standard hydrogen electrode) [8–12]. However, the Zn anode suffers from spongy Zn deposition with uncontrollable dendrite growth. Such loose structure will enhance the chemical corrosion during the repeated platting and stripping processes [13–16]. Unfortunately, the dendritic Zn particles can damage the separator and cause battery short circuits, which hinders the practical application of ZIBs [5, 17–20]. At present, it is urgent to consider strategy to inhibit the growth of Zn dendrite based on the intrinsic mechanism of Zn deposition.

In fact, electrochemical Zn deposition is one of the earliest subjects within the framework of electrochemistry, which takes place at Zn anode and electrolyte interfaces under the influence of an electric field, and includes electrocrystallization [21–25]. The electrocrystallization process usually involves transformation from an unbalanced state to an equilibrium state at a certain overpotential ($\eta$), which can be described as [26]:

1 $$ \Delta G{\text{ }} = {\text{ }} - \frac{{\pi hr^{2} \rho nF\eta }}{A} + 2\pi hr\sigma  $$

where *∆G* is Gibbs free energy of nucleation of Zn electrocrystallization, *h* is the height of Zn atom, *r* is radius of crystal nucleus, *σ* is interfacial tension between electrode and electrolyte, *A* refers to the atomic mass of Zn, *ρ* is the density of nucleus, *n* is valence of Zn^2+^ and *F* is faraday’s constant. The crystal nucleus can exist stably only if *∆G* < 0, otherwise they will dissolve in electrolyte [26, 27]. Correspondingly, the critical size (*r*_*c*_) and Gibbs free energy *∆G*_*c*_ of stable nucleus can be obtained based on ∂*∆G/∂r* = *0:*

2 $$ {\text{r}}_{{\text{c}}} {\text{ = Ah}}\sigma {\text{/}}(\rho {\text{nF}}\eta )  $$

3 $${\Delta }G_{c}  = \pi h\sigma _{{}}^{2} A/(\rho nF{\eta }) $$

**Fig. 1:**
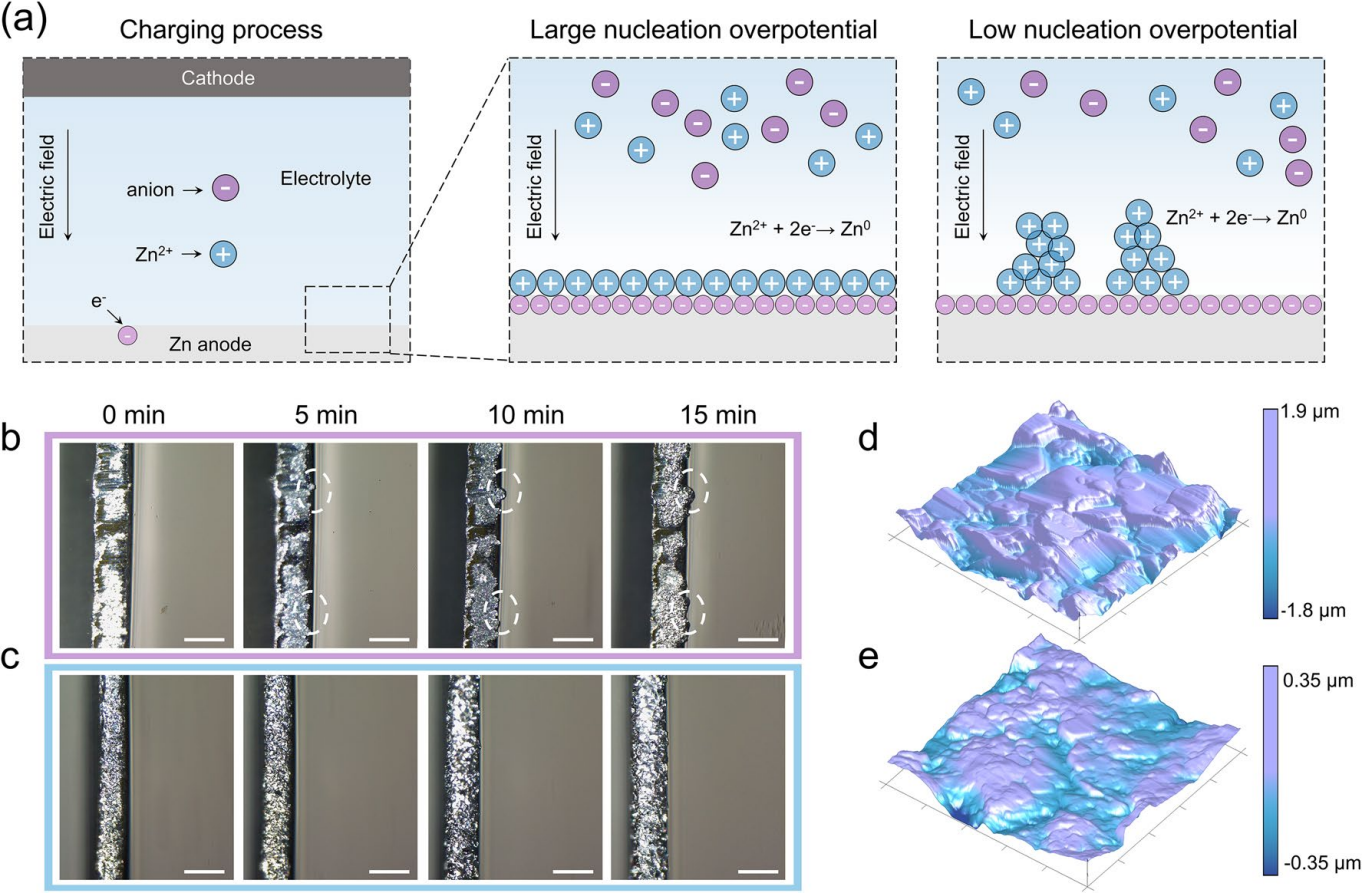
**a** Schematic diagram of the effect of overpotential on the Zn deposition process. **b**–**c** In-situ microscopy images of Zn plating process in the ZS electrolyte and ZS-Na-L electrolyte (Scale bar: 100 μm). **d**–**e** AFM images of the cycled Zn in the ZS electrolyte and ZS-Na-L electrolyte

Obviously, only nuclei with r greater than the *r*_*c*_ can effectively exist and grow [26]. Notably, the* r*_*c*_ is governed by *η*. Figure 1a schematically depicts the relationship between Zn growth behaviors and nucleation overpotential. The higher *η* could drive the smaller* r*_*c*_ of the nucleus, which promotes the growth of metal deposition. In contrast, lower *η* will lead to the deposition of Zn aggregation, resulting in dendrite growth [23, 24, 26]. Additionally, the nucleation rate (ω) could be also calculated using *∆G*_*c*_:

4 $$ \omega  = K\exp [ - \pi h\sigma ^{2} LA/(\rho nF\eta )] $$

where *K* is pre-exponential factor and *L* is Avogadro constant. The equation shows that nucleation rate increases exponentially with the increase of overpotential, allowing for the formation of fine and uniform plating layer. Therefore, the overpotential plays a vital role in achieving uniform deposition of Zn^2+^. In electrocrystallization process, overpotential can arise from two principal causes: on the one hand, it comes from the de-solvation of metal ions in the electrolyte to the surface of the metal electrode (electrochemical overpotential); on the other hand, the hindrance incorporation in the lattice (as result of surface diffusion or displacement of adsorbed species, crystallization overpotential) [22]. Thus, to access dendrite suppressing characteristic, the nucleation overpotential of Zn deposition can be adjusted. Although various strategies have been adopted to design dendrite-free Zn anode, such as surface coating [28], additives in electrolyte [29, 30] and three-dimensional Zn architectures [31, 32], systematic studies on nucleation overpotential of Zn deposition is still rare.

Here, we introduce sodium L-tartrate (Na-L), a typical complexing agent, into ZnSO_4_ (ZS) electrolyte to regulate nucleation overpotential for dendrite-free ZIBs. The experiments and theoretical simulations revealed that L-Tartaric anions (L^−^) could enter the primary solvation shell of Zn^2+^, decreasing the number of H_2_O molecules and increasing de-solvation energy barrier suitably. Besides, Na^+^ can be preferentially adsorbed on Zn metal anode and induced a dynamic electrostatic shielding layer around abrupt Zn protuberance, which hinders the simple agglomerative Zn deposition [33, 34]. Under the effect of Na-L additive, the Zn nucleation overpotential in modified electrolyte (ZS-Na-L) could increase from 28.3 to 45.9 mV. Consequently, the ZS-Na-L electrolyte enables Zn-Cu cells to deliver a high Coulombic efficiency (CE) of ~ 99.8% for 600 cycles at a current density of 10 mA cm^−2^, and long-term cycling stability of Zn-Zn cells up to 1500 h. At a current density of 10 mA cm^−2^, the Zn-Zn cell with optimized electrolyte exhibited a stable Zn deposition for ~ 500 h. Moreover, the Zn full cells with high mass loading Li_2_MnO_4_ (LMO) cathode (~ 12 mg cm^−2^) deliver a stable discharge capacity of 90 mAh g^−1^ after 500 cycles. The results demonstrate the importance of nucleation overpotential on Zn deposition and provide a research paradigm for other metal anodes.

## Experimental and Calculation

### Preparation of Electrolyte

The 2 M ZS electrolyte was prepared by dissolving 1 mol of ZnSO_4_·7H_2_O (99.995%, Aladdin) in deionized (DI) water to acquire 0.5 L of solution. The ZS-Na-L electrolytes were prepared by adding different amounts (2, 4, and 6 mmol) of sodium L-tartrate (≥ 98%, Aladdin) into 100 mL 2 M ZS electrolyte.

### Preparation of Electrodes

The purchased Zn foil (99.99%) was polished with sandpaper and punched into a disc to be employed as Zn anode. The LMO cathode was obtained through mixing 70 wt% LMO powders (Hefei kejing Co., Ltd.), 20 wt% conductive carbon (KS-6, MTI, Co. Ltd.) and 10 wt% polytetrafluoroethylene (PTFE) in isopropanol (Sigma-Aldrich Co., Ltd.). The obtained slurry was coated on the Ti mesh and dried at 60 °C for 12 h under vacuum. The mass loading of active material on the Ti mesh was about 12 mg cm^−2^.

### Materials Characterizations

Hitachi SU-70 field-emission SEM was employed to investigate morphology for Zn foil and LMO. The phase structure of materials was explored using D8-XRD (Bruker AXS, WI, USA). To characterize the interface morphologies of Zn plating process, the optical microscope (Leica DVM6) was employed. AFM images were collected by Icon (Bruker) AFM. The ATR-FTIR spectra were conducted by Bruker Alpha FT-IR spectrometer. *In-situ* differential electrochemical mass spectrometry was performed on a commercial mass spectrometer (Hiden, Beijing) with a Zn-Zn cell containing Zn electrodes, glass fiber separator, and a stainless-steel spacer (height: 1 mm). Before testing, the system was deflated with Ar for 12 h (~ 5 × 10^6^ Torr), and then the resulting gas was used during 5 mA cm^−2^ charging/discharging. The Raman analysis was done by DXR Raman microscope with an excitation length of 532 nm.

### Electrochemical Characterization

Zn foils with a thickness of 100 μm as the electrode, 100 μL electrolyte and a piece of glass fiber (GE-Whatman) separator were assembled in Coin-type (CR 2032) cells. 50 μm Zn foil was employed for high ZUR test. After polished, the mass loading is ~ 30 mg cm^−2^, corresponding to theoretical capacities of ~ 25 mAh cm^−2^. Zn-LMO full cells with ~ 10 μm Zn foils (at the N/P ratio of ~ 4.2, ~ 24% ZUR) were tested in different electrolytes. The Galvanostatic charge and discharge measurements were conducted on a Neware battery systems instrument (CT-4008 T) after resting for 4 h. CV, LSV and EIS tests were performed by a CHI 660e electrochemical workstation (ChenHua Instruments Co.). The corrosion behavior was performed using three-electrode system (Zn foil as working electrode, Pt as the counter electrode, and Ag/AgCl as reference electrode) on the CorrTest CS2350H electrochemical workstation (Wuhan CorrTest Instrument Corp., Ltd. China).

### Finite Element Method Simulations

The modeling of Zn electrodeposition was simulated by using COMSOL Multiphysics 6.0. The height of the simulation area is 6 μm and the width is 10 μm. The mesh is selected based on triangles or tetrahedra, while using progressively fine refinements for the electrode bands (Fig. S17). The Zn deposition process is described by Butler-Volmer equation. Furthermore, the Butler-Volmer equation must account for the ion concentration on the surface since Na^+^ adsorbs on the surface in ZS-Na-L electrolyte.

5 $$ {\text{i}}_{{{\text{loc}}}}  =  - (C^{\prime} - k\theta )\exp \left(\frac{{ - (\alpha  - k^{\prime}\theta )F\eta }}{{RT}}\right)i_{0}  $$

where θ is the coverage of adsorbed inhibiting additive and cannot exceed unity, C’ is the coefficient of Zn^2+^ concentration, k is coefficient of Na^+^, k’ is the inhibiting transfer coefficient of the Na^+^, F is the Faraday constant, R is the molar gas constant, T is the ambient temperature, η is the overpotential, i_0_ is the exchange current density. Before simulation, a semi ellipse nucleus was set.

### Computational Details

Molecular dynamics (MD) simulations were conducted in the Forcite module in Materials Studio of Accelrys Inc. A condensed phase optimized molecular potentials for atomistic simulation studies (COMPASS II) and force field were chosen for all molecular dynamics simulations, and the time step was fixed to be 1 fs. The size of box is 5 nm × 5 nm × 5 nm. The simulation cells contained 3055 H_2_O, 200 ZnSO_4_ and 4 Na-L. The electrolyte systems were equilibrated in the isothermal-isobaric ensemble (NPT) with a pressure of 0.1 GPa and a decay constant of 0.1 ps for 500 ps. The temperature was set to be 298 and 323 K with a Nose thermostat. Next, another 1000 ps simulation operation was performed in the Canonical Ensemble (NVT). The simulation time was long enough to ensure that the equilibrium states of the electrolyte systems were reached.

The density function theory (DFT) was performed using generalized gradient approximation (GGA) and Perdrew-Burke-Ernzerhof (PBE) exchange correlation functional in Castep module of the Materials Studio of Accelrys Inc. The cutoff energy with the value of 800 eV was used in all the calculations [35–38]. The Γ point was set for saving the computational resources. The convergence criterion for the electronic structure iteration was set to be 10^−5^ eV, and that for geometry optimizations was set to be 0.02 eV Å^−1^ on force. Zn (002) surface is modeled by 4 layers 5 × 5 supercell and a vacuum thickness of 15 Å is applied. The bottom two layers were kept fixed to maintain bulk property.

The quantum chemical computations for water molecules and ions were carried out on DMOl3 package in Materials Studio at the level of DFT. Geometry optimizations and energy calculations were performed using GGA and PBE. The energy convergence criterion was set to be 10^–6^ Hartree. The binding energy (*E*_b_) is defined as following:

6 $$ E_{{\text{b}}} = E_{{{\text{total}}}} - E_{{\text{c}}} - E_{{\text{m}}} $$

where, *E*_total_ is the total energy of the system, *E*_c_ is the energy of cation and *E*_m_ is the energy of water molecules.

## Results and Discussion

### Morphology of Zn Deposition Using Different Electrolytes

The Na-L modified electrolyte (ZS-Na-L) was prepared via dissolving Na-L into ZS electrolyte. To explore the effect of Na-L on Zn^2+^ plating behavior, *in-situ* optical microscopy was employed to visually scrutinize the surface morphology evolution of Zn electrodes at current density of 10 mA cm^−2^. As presented in Fig. 1b, the Zn electrode in ZS electrolyte displays some bubbles on the surface at approximately 5 min, which continue to grow and eventually transform into dendrites at 15 min. In contrast, the Zn deposition process is homogeneous and stable using the ZS-Na-L electrolyte (Fig. 1c). After 15 min plating, no dendrite-like morphology is observed, indicating that the Na-L is advantageous for homogenizing the nucleation sites and inhibiting the growth of dendrites. The surface features of Zn electrode cycled in different electrolytes were also characterized using atomic force microscopy (AFM), as illustrated in Fig. 1d, e. The Zn electrode cycled in electrolyte with Na- L additive has a much smooth surface with height of ~ 0.35 μm. However, Zn electrode cycled in the ZS electrolyte possesses a rough surface with the height of ~ 1.9 μm. Such a high surface roughness can be attributed to the inhomogeneous deposition of Zn^2+^. Figure S1 exhibits the scanning electron microscopy (SEM) images of the cycled Zn electrode. The Zn surface in ZS electrolytes becomes rough with flake-like dendrites due to the continuous reactions. In comparison, the Zn electrode cycled in the ZS-Na-L electrolyte shows a dense and smooth morphology, further demonstrating the promising regulation of Zn deposition behavior by Na-L.

### Physicochemical Investigation on the Role of Na-L for Zn Deposition

**Fig. 2:**
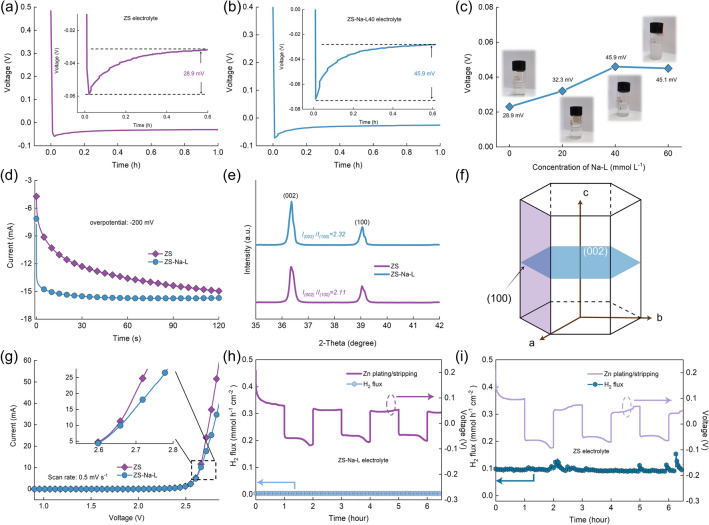
The nucleation overpotential of Zn plating with **a** ZS electrolyte, **b** ZS-NA-L 40 electrolyte; **c** Concentration of Na-L versus potential curves. Inset images are the optical images of ZS electrolyte with different concentrations of Na-L. **d** CA curves of Zn-Zn cells using different electrolytes at an overpotential of -150 mV; **e** XRD pattern of Zn deposits from the Zn electrodes of Zn-Zn cell under a current density of 2 mA cm^−2^ for 1 h. **f** Illustration of hexagonal structure of Zn. **g** Linear sweep voltammetry curves of Zn-Ti cell in different electrolytes at a scan rate of 1 mV s^−1^. In situ DEMS curves of Zn-Zn cells in **h** ZS-Na-L electrolyte and **i** ZS electrolyte

To verify the effect of Na-L additive on the nucleation overpotential, ZS electrolytes containing different concentrations of Na-L were prepared. As shown in Fig. 2a, the overpotential in ZS electrolyte exhibits only ~ 28.9 mV. After introducing 20 mmol L^−1^ Na-L into ZS electrolyte (ZS-Na-L20), the Zn-Cu cell delivers an overpotential of ~ 32.3 mV (Fig. 2b). When the Na-L concentration was further increased to 40 and 60 mmol L^−1^, overpotential increased to 45.9 and 45.1 mV respectively (Figs. 2c and S2). This result demonstrates that the nucleation overpotential could rise with the increasing Na-L concentration, but the overpotential is essentially stable at a concentration of 40 mmol L^−1^ or higher. From optical images of electrolytes with different concentrations of Na-L (Fig. 2c), ZS-Na-L40 is homogeneous and clear. However, ZS-Na-L60 is suspensive due to the recrystallization of the solute. Hence, 2 M ZnSO_4_ with 40 mmol L^−1^ Na-L is optimal. According to Eq. 1, the Δ*G* and *r*_c_ also related to interfacial tension in addition to overpotential. The contact angle measurements were conducted. The almost unchanged contact angle demonstrate Na-L has no effect on interfacial tension between Zn electrode and electrolyte (Fig. S3). The pH and ionic conductivity are also key parameters in electrolyte. As shown in Figs. S4-S5, with the increase of additive concentration, the ionic conductivity and pH value of the electrolyte will increase. Therefore, the Na-L additive is effective for ion transport and inhibition of hydrogen evolution reaction. The Zn deposition mechanism in ZS and ZS-Na-L electrolytes was explored by chronoamperometry (CA) test (Fig. 2d). The continuous increasing current density in ZS electrolyte indicates a rampant two-dimensional (2D) diffusion and uneven dendrite growth due to tip effect [39, 40]. In contrast, the current density in a cell containing ZS-Na-L electrolyte stabilizes immediately after applying an overpotential, indicating three-dimensional (3D) diffusion of a uniform crystal [41–43]. Moreover, to evaluate the reversibility, Zn-Ti cell was assembled to investigate Zn plating/stripping behaviors. As shown in cyclic voltammetry (CV) curves in Fig. S6, the Zn nucleation in ZS-Na-L electrolyte exhibits larger polarization voltage than that in ZS electrolyte (96 mV), indicating improved driving force for nucleation at the initial Zn deposition in ZS-Na-L electrolyte [44, 45].

The crystal structure of Zn deposition acquired from different electrolytes was collected by X-ray Diffraction (XRD). As a result, a stronger (002) peak could be seen in ZS-Na-L electrolyte, as shown in Fig. 2e. The enlarged (002) planes for Zn deposition in ZS-Na-L electrolyte are demonstrated quantitatively by the increase in *I*_(002)_/*I*_(100)_ from 2.11 to 2.32. Based on epitaxial mechanism that drives the surface texture with the crystallographic orientation of the (002) plane (Fig. 2f), it is safe to conclude that the as obtained surface of the (002) crystal plane was smooth and conductive to uniform Zn^2+^ deposition [40, 46]. To analyze the inhibition effect of Na-L on parasitic reaction between Zn electrodes and electrolyte, the linear sweep voltammetry (LSV) and linear polarization test were employed. As presented in Fig. 2g, the LSV curves exhibit a much higher oxygen evolution reaction overpotential of the cell with ZS-Na-L electrolyte. From the linear polarization curves in Fig. S7, increased corrosion potential could be realized using Na-L, indicating a lower tendency of corrosion of Zn electrodes. To accurately quantitate gas production, *in-situ* differential electrochemical mass spectrometry (DEMS) was set up to detect hydrogen flux during Zn striping/plating process in different electrolytes at a current density of 5 mA cm^−2^. As shown in Fig. 2h, the hydrogen evolution rate during Zn-Zn cells cycling in ZS electrolyte could reach from initial 0.1–0.15 mmol h^−1^ cm^−2^. Inversely, there is almost no H_2_ detected in ZS-Na-L electrolyte, indicating that the Na-L additive successfully modulated the side reaction.

### Intrinsic Mechanism of Na-L Increased Overpotential

**Fig. 3:**
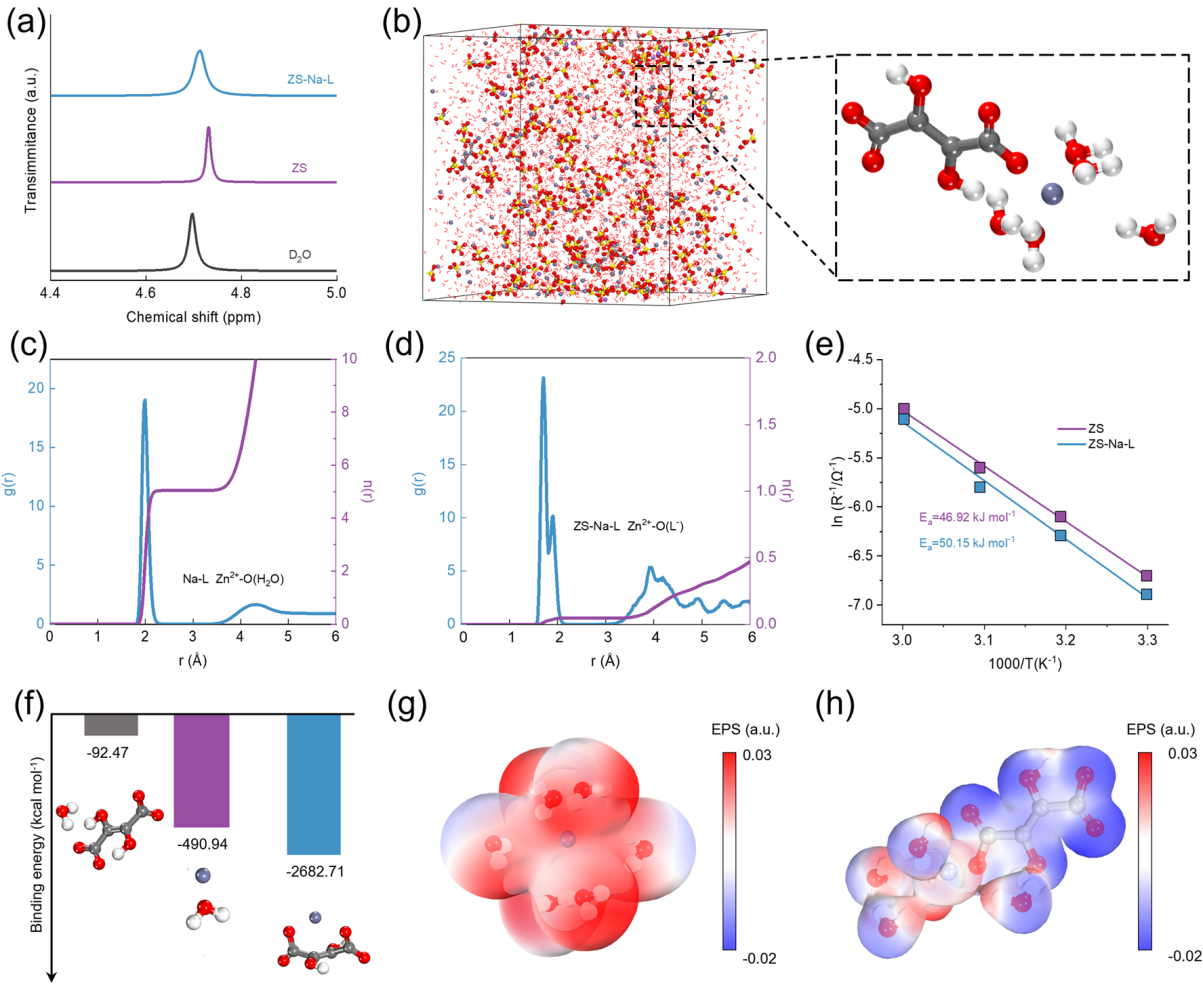
**a** ^2^H spectra of H_2_O from D_2_O, ZS and ZS-Na-L. **b** Snapshot of ZS-Na-L system obtained from MD simulation and partial enlarged snapshot referring to Zn^2+^ solvation structure. **c** RDFs for ZS-Na-L Zn^2+^-O (H_2_O) and **d** Zn^2+^-O (Na-L) collected from MD simulations in ZS-Na-L system. **e** Calculated activation energies in ZS and ZS-Na-L electrolytes. **f** Binding energy for Zn^2+^ with different compounds (H_2_O, L^−^) under DFT calculations. **g-h** Electrostatic potential mapping of the original Zn^2+^-6H_2_O (left) and Zn^2+^-5H_2_O-L^−^ (right) solvation structures

To investigate the unique role of Na-L in the electrolyte, a series of characterizations including nuclear magnetic resonance (NMR), Raman and Fourier transform infrared spectroscopy (FTIR) were conducted. Figure 3a shows the ^2^H NMR spectra of pure D_2_O, ZS electrolyte and ZS-Na-L electrolyte. ^2^H peak shift increases from 4.69 to 4.73 ppm when ZS is added into Pure D_2_O, implying a decreased surrounding electronic density, and weakened shielding of proton in water molecules, denoting less free water in ZS environment due to the strong coordination between Zn^2+^ and D_2_O [47, 48]. When compared to the ZS electrolyte, the ^2^H peak in the ZS-Na-L electrolyte shifts lower to 4.71 ppm, indicating that more H_2_O molecules was in a free state [49]. Such results demonstrate that the addition of Na-L has a function of weakening the solvation interaction between Zn^2+^ and H_2_O, which can also be confirmed by FTIR test (Fig. S8). The stretching of SO_4_^2−^ in ZS electrolyte shifts to higher wavenumbers after introducing Na-L. Raman spectroscopy also revealed that SO_4_^2−^ band exhibited stronger shoulder shift to low frequency with increasing Na-L increasing (Fig. S9). Such results indicate that the introduction of Na-L impairs the electrostatic coupling between Zn^2+^ and SO_4_^2−^ and weakens the constraint around SO_4_^2−^, thus further confirming the regulated Zn^2+^ solvation structure [48].

The solvation structure of Zn^2+^ in different electrolytes was further analyzed by molecular dynamics (MD) simulations. The statistical results of MD simulations in Fig. S10 show that the primary solvation shell (PSS) of Zn^2+^ consists of six H_2_O molecules in ZS electrolyte. In comparison, a new solvation structure, with five H_2_O and one L^−^ was observed in ZS-Na-L electrolyte, demonstrating an obvious change in solvation structure after introducing Na-L (Fig. 3b). To quantitatively investigate the solvation structure, radial distribution functions (RDFs) and coordination number (CN) analysis were carried out. For ZS electrolyte, the first solvation shell of Zn^2+^ is represented by the first RDF peak, which locates at ~ 2 Å and corresponds to Zn-OH pairs with CN of ~ 5.3 (Fig. S11). For Zn-Na-L electrolyte, the first RDF peak of Zn-L is located at ~ 1.8 Å, indicating the involvement of Na-L in the first solvation structure of Zn^2+^ (Fig. 3c). Correspondingly, Fig. 3d shows that the CN of Zn-OH in ZS-Na-L electrolyte decreases from 5.3 to 4.72, indicating that the solvation structure is significantly changed by Na-L. As shown in Fig. S12, the number of hydrogen bonds in ZS-Na-L electrolyte is lower than that of ZS system, demonstrating that the Na-L is favorable of destructing original hydrogen-bond network inside pure ZS environment since they can push H_2_O molecule out of PSS. The energy barrier in the de-solvation process of Zn^2+^ was quantitatively evaluated from activation energy (*E*_*a*_) via law of Arrhenius [50]. The Nyquist plots of Zn-Zn cells at different temperatures and the charge transfer resistance (*R*_*ct*_) could be easily acquired (Fig. S13 and Table S1). The *E*_*a*_ was calculated by fitting ln(1/*R*_*ct*_) vs. 1000/T in different electrolytes [51, 52]. As shown in Fig. 3e, the *E*_*a*_ is 46.92 kJ mol^−1^ in ZS electrolyte, whereas the *E*_*a*_ in ZS-Na-L electrolyte is higher (50.15 kJ mol^−1^). Such results demonstrate that Na-L additive could bring higher de-solvation energy barrier in ZS-Na-L electrolyte, which contributes the improved nucleation overpotential. To analyze the transportation capability of different electrolytes, the mean-squared displacement (MSD) versus time were employed (Fig. S14). The diffusion coefficient of Zn^2+^ increases based on the slope (MSD vs. time) when Na-L is added in ZS electrolyte, revealing Na-L is helpful for transferring Zn^2+^ to some extent. To further understand the impact of Na-L addition, quantum chemistry calculations were performed. Compared with the interaction between H_2_O and L^−^, it is easier for Zn^2+^ to bind with H_2_O and L^−^, and obviously Zn^2+^ is much more inclined to combine with L^−^ than H_2_O (Fig. 3f), in accordance with the solvation structure from MD simulations. Meanwhile, the electrostatic potential of Zn^2+^ solvation structure was observed in Fig. 3g. The electrostatic potential decreased when one L^−^ is introduced into original Zn^2+^-6H_2_O PSS to replace one of the H_2_O, indicating that the electrostatic repulsion around Zn^2+^ can be weakened and beneficial for the fast transportation.

**Fig. 4:**
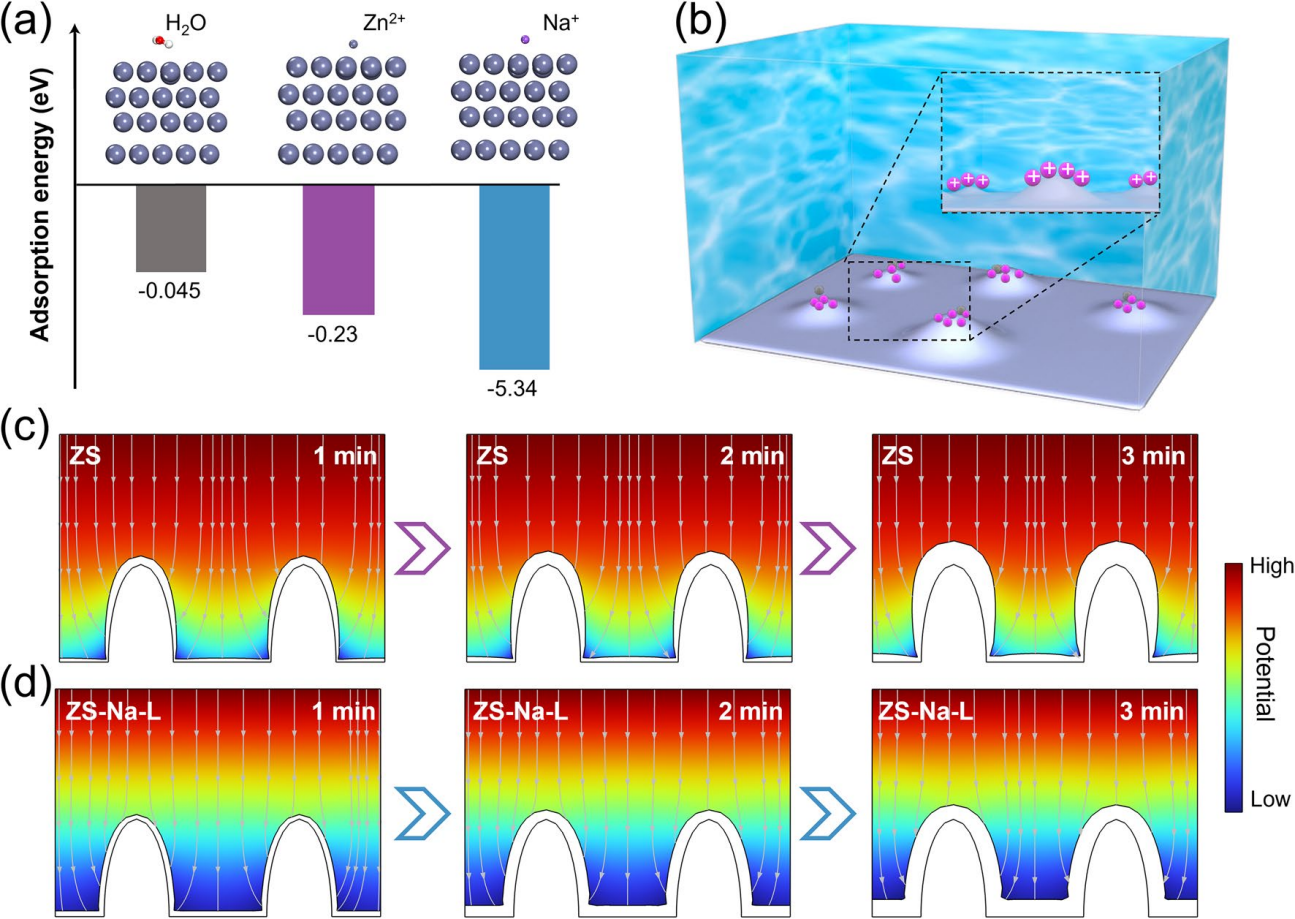
**a** Adsorption energies of H_2_O, Na^+^, and Zn^2+^ on Zn (001) surface. **b** Schematic Diagrams of the role of Na^+^. FEM simulations results of the Zn−electrolyte interface at 1, 2 and 3 min (from left to right) in **c** ZS and **d** ZS-Na-L electrolytes (Up: cathode, down: anode). The black lines show the original Zn-electrolyte interface

Considering that Zn plating/stripping occurs at the interface, the interacting behavior between Zn anode and electrolyte was further studied. We first carried out density functional theory (DFT) calculations to the adsorption energy between Zn slab and different molecules was compared. As shown in Fig. 4a, the adsorption energy of Na^+^ is much lower than that of H_2_O, L^−^ or Zn^2+^ on Zn (002) crystal plane, indicating that the Na^+^ preferentially adsorbs on Zn surface instead of the other molecules and thus acts as a dendrite inhibitor (Fig. 4b). These can also be supported by electrochemical test results. The Nyquist plots of Zn-Zn cells in ZS-Na-L electrolyte exhibit higher charge transfer resistance (Fig. S15), indicating the adsorption of Na^+^ on the surface of electrode. Additionally, less Zn^2+^ electrostatically interacts with Zn metal in ZS-Na-L electrolyte, which is evidenced by the zeta potential test (Fig. S16) [34, 53, 54].

To further reveal the impact of Na^+^ before adsorption on the Zn deposition, surface evolution on Zn foils was explored by finite element method (FEM) simulations. Detailed parameters are provided in Experiment section. As exhibited in Fig. 4c, for ZS electrolyte, current density at tip of the initial stage is higher than the other regions. As the plating time increases from 1 to 3 min, gradual and continuous deposition of Zn tends to occur on the uneven surface, eventually leading to a severe problem of uncontrollable growth. Conversely, for ZS-Na-L electrolyte, the Na^+^ will be absorbed on the surface of Zn electrode before the dendrite growth and shielding the cutting-edge electric field [55]. Finally, the more Zn will grow smoothly with reduced dendrite growth (Fig. 4d), which is also consistent with the above experimental observation of optical image and AFM (Figs. 1c and S1).

### Electrochemical Performance of Zn Anode with Different Electrolytes

**Fig. 5:**
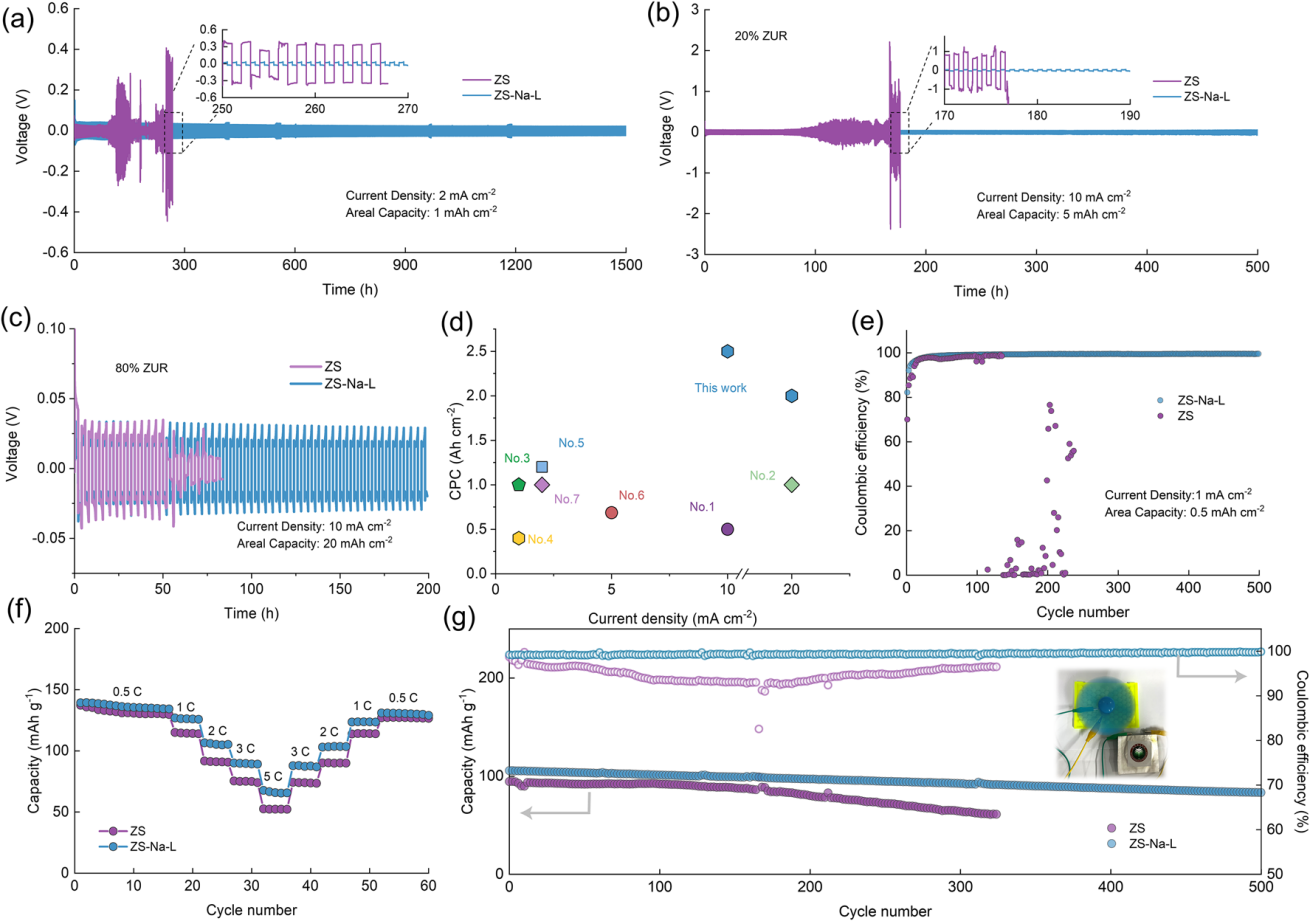
Cycling performance of Zn-Zn symmetric cells with or without Na-L addition collected at **a** 2 mA cm^−2^, 1 mAh cm^−2^ and **b** 10 mA cm^−2^ and 5 mAh cm^−2^. **c** 10 mA cm^−2^ and 20 mAh cm^−2^ respectively. **d**. CPC comparison of Zn-Zn cell between this work and other reports. The detailed references corresponding to the point number are listed in Table S2. **e** Zn plating/stripping CE at 1 mA cm^−2^ and 0.5 mAh cm^−2^ in different electrolytes. **f** Rate performance and **g** cyclic stability and efficiency of Zn-LMO cells in two electrolytes at 2C, inset image showing Zn-LMO cell drives a fan

The uniform Zn deposition could be achieved with the help Na-L additive through previous experimental representation and theoretical analysis. Therefore, it is expected that the electrochemical performance of Zn anodes could be greatly improved. Certainly, these results demonstrate that the Na-L additive is conductive to homogeneous Zn deposition. To confirm this speculation, we firstly investigated the rate performance of symmetric cells with a fixed capacity of 1 mAh cm^−2^ at various current densities from 1 to 10 mA cm^−2^ (Figs. S18–S19). The Zn-Zn cells tested in ZS-Na-L electrolyte exhibit more stable voltage profile than that in ZS electrolyte. Cycling performance of Zn-Zn cells was also evaluated at different current densities and areal capacities. As shown in Fig. 5a, after introducing Na-L into ZS electrolyte, the Zn-Zn cells can achieve a long cycle lifespan of 1500 h at 2 mA cm^−2^ with a capacity per cycle of 1 mAh cm^−2^, but cells in ZS electrolyte exhibit inferior cycle stability of 120 h. To test Zn utilization rate (ZUR) in different electrolyte, Zn foil with mass loading ~ 30 mg cm^−2^, corresponding to theoretical capacities of ~ 25 mAh cm^−2^, were employed. As shown in Fig. 5b, the excellent stability of 500 h can also be obtained in the presence of Na-L with a high current density of 10 mA cm^−2^ and ZUR of 20% (5 mAh cm^−2^). In contrast, the Zn-Zn cells in ZS electrolyte cause short circuiting after only 120 h. Further, we tested Zn-Zn cells at a high ZUR of 80% (20 mAh cm^−2^). The symmetric cell in ZS-Na-L electrolyte could be cycled stably for 200 h, which is 4 times that of the cell in ZSO electrolyte. Excitingly, the cumulative plated capacity (CPC) in this work are superior to other recent reports as depicted in Fig. 5d, suggesting the advantage of increasing overpoetntial strategy in protecting Zn anodes. The reversibility of Zn plating/stripping chemistry was further investigated using Zn-Cu cell. Figure 5e shows the CE of Zn anodes in different electrolytes. Obviously, the CE is rather low in the initial cycles. This is because the deposition process of Zn on Cu encounters a reshaped Zn coordination. After that, the CE drastically fluctuates and decreases only after 110 cycles due to the dendrite growth and side reactions. But for Zn-Cu cells using ZS-Na-L electrolyte, although the initial CE is close to that of ZS electrolyte, it achieves stability and high stripping/plating efficiency with a remarkable average CE of 99.7% after 500 cycles. The galvanostatic charging/discharging curves of Zn-Zn cells were further measured (Fig. S20). A small voltage platform could be observed in charging process, attributing to the Zn-Cu de-alloying [56]. Even under high current densities of 10 mA cm^−2^, the Zn electrodes in ZS-Na-L electrolyte also demonstrated better reversibility than ZS electrolyte (Fig. S21).

Considering electrolyte is necessary to energy storage systems [57, 58], the electrochemical properties of Zn full cell are evaluated with commercial LiMn_2_O_4_ (LMO) as cathode material. Notably, 1 M Li_2_SO_4_ was added into both ZS and ZS-Na-L electrolyte to provide the lithium sources during the full cell test. The octahedron morphology of LMO is shown in Fig. S22. The XRD pattern in Fig. S23 exhibits the pure phase of LMO. The reaction kinetics of Zn-LMO cell based on ZS-Na-L electrolyte were investigated under different scanning rates (Fig. S24). Based on equation of *i* = *av*^*b*^, the fitted *b* values for redox peak are 0.65 and 0.72, indicating the combined contribution by diffusion-controlled and capacitive reactions [59]. The rate performance of the Zn-LMO cell with different electrolytes was explored at various current densities as shown in Figs. 5f and S25. In the ZS-Na-L electrolyte, a significantly enhanced rate capacity can be achieved. The capacity for ZS-Na-L electrolyte is larger than that for ZS electrolyte with further cycling under current densities of 0.5C, 1C, 2C, 3C and 5C, demonstrating the effective role of Na-L additive. The cycle stability of Zn-LMO cell using ZS and ZS-Na-L electrolytes is further investigated at the current density of 2 C. Inset image in Fig. 5g exhibits a fan can be driven by a LMO full cell. The capacity retention of both cells could reach ~ 99.3% at the first 100 cycles. However, Zn-LMO cell in ZS electrolyte cannot operate properly after 300 cycles, which could be attributed to the dendrite formation and side reactions (Fig. S26). In comparison, the cell with ZS-Na-L electrolyte can retain a reversible capacity of 90 mAh g^−1^ with a high CE of 99.7% after 500 cycles, further demonstrating the effective role of Na-L.

## Conclusions

In summary, we introduced a green and economic Na-L electrolyte additive to regulate the thermodynamic behavior of Zn deposition. MD simulations combined with experimental studies confirmed the strong interaction between L^−^ and Zn^2+^, modulating Zn^2+^ solvation structure and increasing de-solvation energy barrier. Additionally, Na^+^ preferentially absorbs on the Zn metal surface, preventing the deposition of Zn^2+^ aggregation. Both effects improve the nucleation overpotential of Zn deposition and assist in reducing the uncontrollable growth of dendrite, which can be observed in optical microscope and AFM images. As a result, dendrite-free and intrinsically stable Zn plating/stripping can be realized in the electrolyte with Na-L. Moreover, Zn-LMO cells using ZS-Na-L electrolyte deliver high levels of capacity, CE, and stability, demonstrating a significant impact of nucleation overpotential on performance of ZIBs. Additionally, we believe that other complexing agents such as amino carboxylate, organic phosphonate and phosphate can also be employed to regulate nucleation overpotential for the development of advanced energy storage devices.

### Supplementary Information

Below is the link to the electronic supplementary material.

Supplementary file1 (PDF 1323 KB)
