# Supplementary material for: Highly Reversible Zn Metal Anodes Enabled by Increased Nucleation Overpotential
Source: Nanomicro Lett. 2023 Jul 6;15:171. doi: 10.1007/s40820-023-01136-z (PMC10326211; doi:10.1007/s40820-023-01136-z)
Supplement: Supplementary file 1 — Supplementary file1 (PDF 1323 KB) [file 40820_2023_1136_MOESM1_ESM.pdf]

Supporting Information for

Highly Reversible Zn Metal Anodes Enabled by Increased Nucleation Overpotential

Zhengqiang Hu[1], Fengling Zhang[1], Anbin Zhou[1], Xin Hu[1], Qiaoyi Yan[1], Yuhao Liu[1], Faiza Arshad[1], Zhujie Li[3], Renjie Chen[1, 2, 3, *], Feng Wu[1, 2, 3], Li Li[1, 2, 3, *]

[1]Beijing Key Laboratory of Environmental Science and Engineering, School of Materials Science & Engineering, Beijing Institute of Technology, Beijing 100081, P. R. China

[2]Collaborative Innovation Center of Electric Vehicles in Beijing, Beijing 100081, P. R. China

[3]Advanced Technology Research Institute, Beijing Institute of Technology, Jinan 250300, P. R. China

*Corresponding authors. E-mail: lily863@bit.edu.cn (Li Li); chenrj@bit.edu.cn (Renjie Chen)

Supplementary Figures and Tables

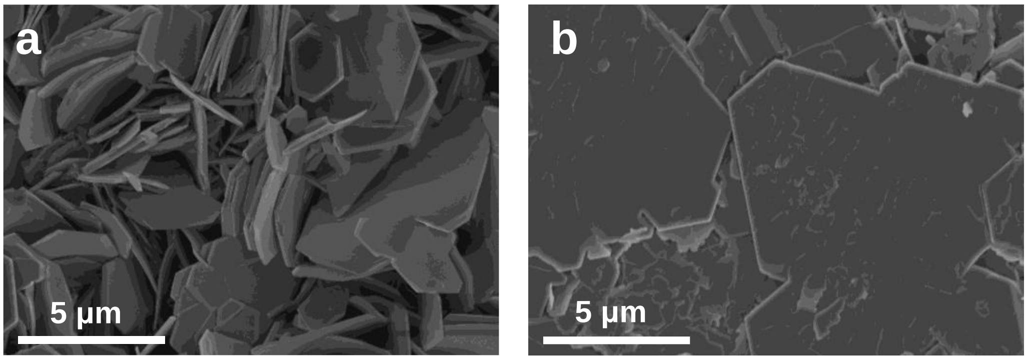

Fig. S1 SEM images of Zn electrode surface after cycling with **a** ZS electrolyte, **b** ZS-Na-L electrolyte

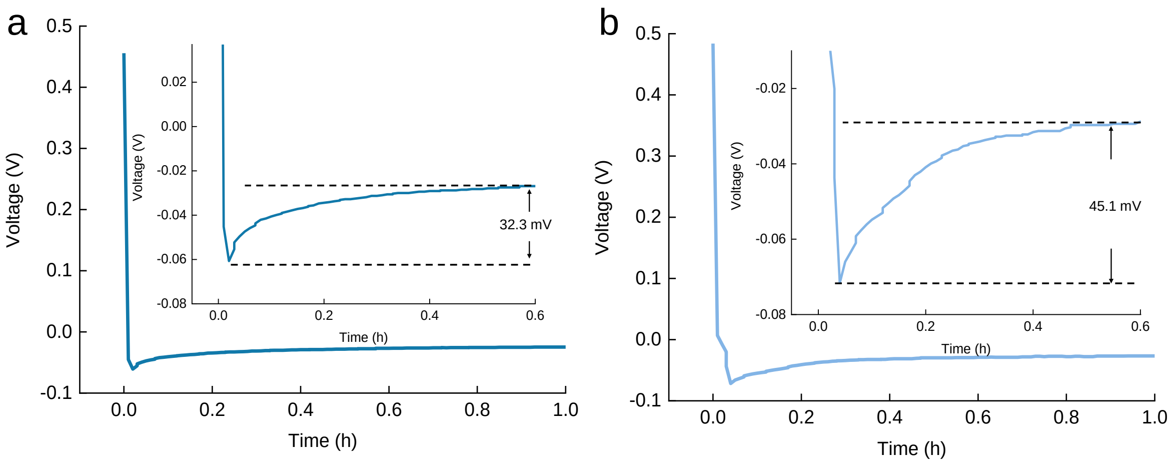

Fig. S2 The nucleation overpotential of Zn plating with **a** ZS-Na-L20 electrolyte and **b** ZS-Na-L60 electrolyte

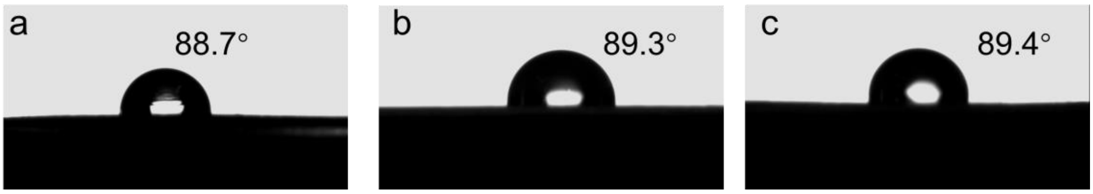

Fig. S3 Contact angle of different electrolytes **a** ZS; **b** ZS-Na-L20; **c** ZS-Na-L40

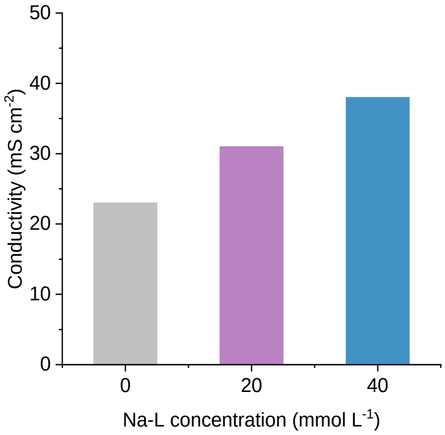

Fig. S4 Ionic conductivity of electrolytes with different Na-L concentrations

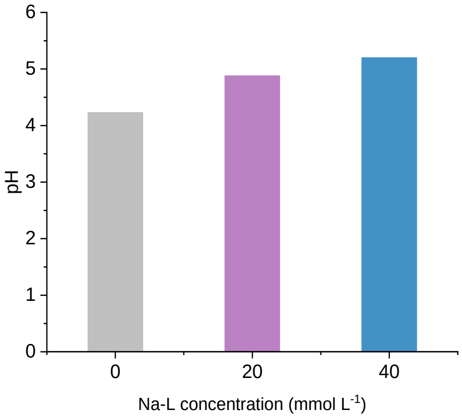

Fig. S5 pH value of electrolytes with different Na-L concentrations

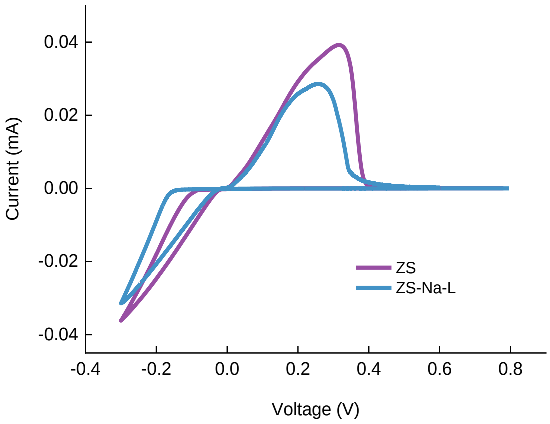

Fig. S6 Cyclic voltammograms (CV) of Zn-Ti cells in ZS and ZS-Na-L electrolytes

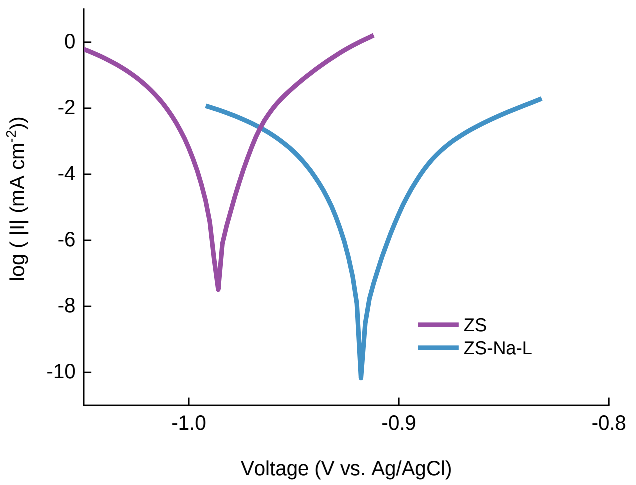

Fig. S7 Linear polarization curves of the fresh Zn electrodes collected with a scanning rate of 0.1 mVs^{-1} in ZS and ZS-Na-L electrolytes using a three-electrode cell

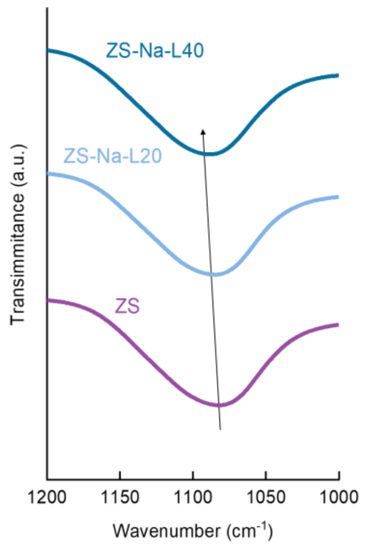

Fig. S8 FTIR spectra for ZS, ZS-Na-L20 and ZS-Na-L40 electrolytes

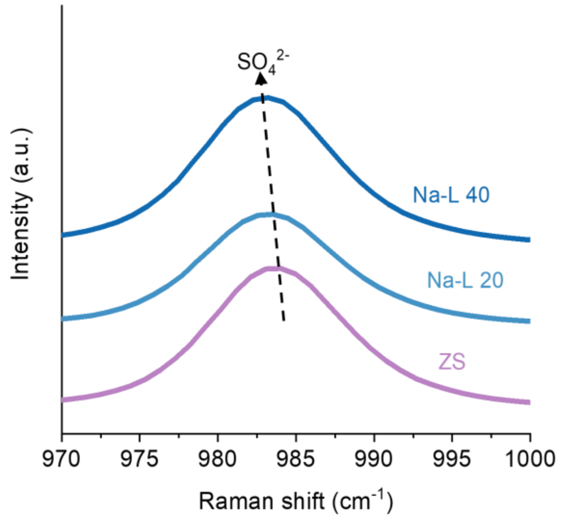

Fig. S9 Raman spectra of electrolyte with different concentrations of Na-L

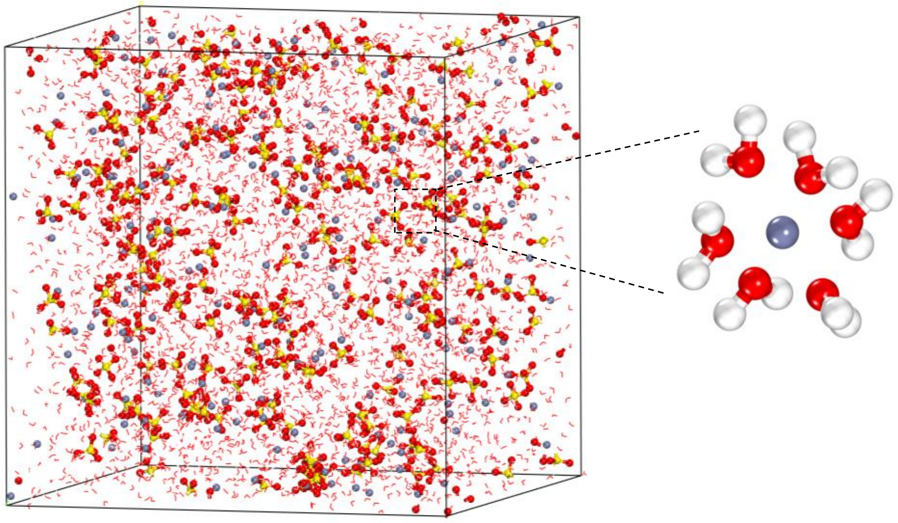

Fig. S10 3D snapshot of ZS electrolyte system obtained from MD simulations and partial enlarged snapshot representing Zn^{2+} solvation structure in ZS electrolyte

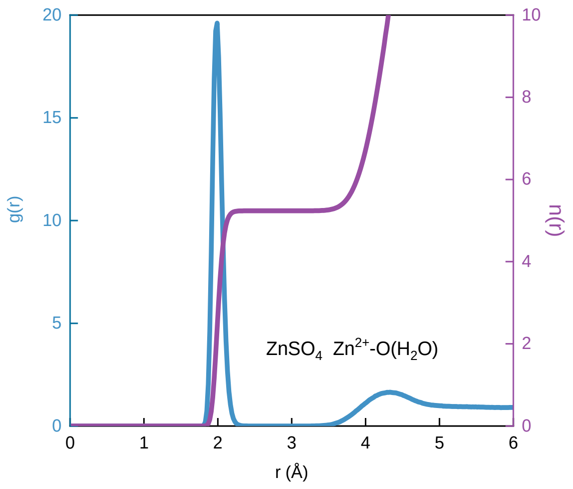

Fig. S11 RDFs for Zn^{2+}-O (H_2O) collected from MD simulations in ZS electrolyte

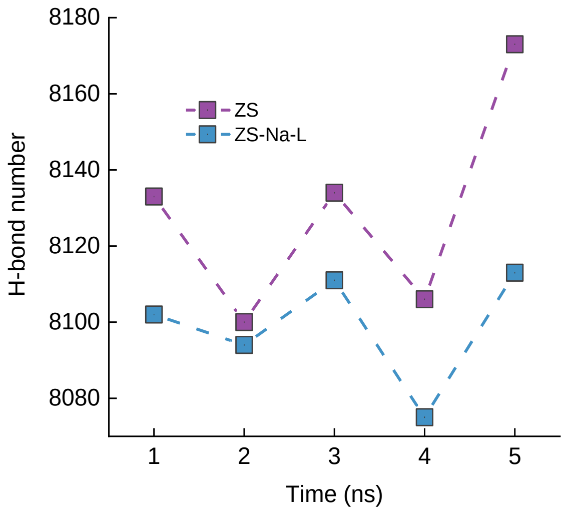

Fig. S12 Number counts for hydrogen-bonds inside ZS and ZS-Na-L electrolyte

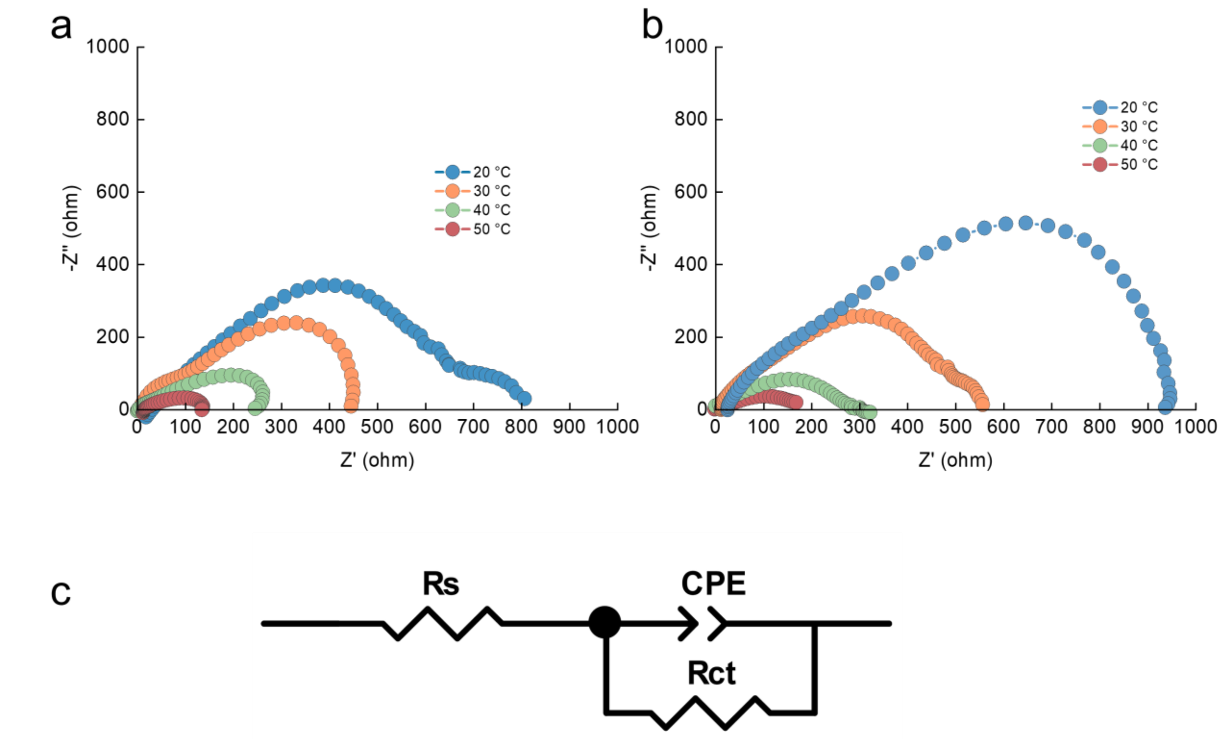

Fig. S13 EIS spectra of Zn-Zn cells at different temperatures in **a** ZS and **b** ZS-Na-L electrolytes. **c** Fitting circuit of symmetric cells

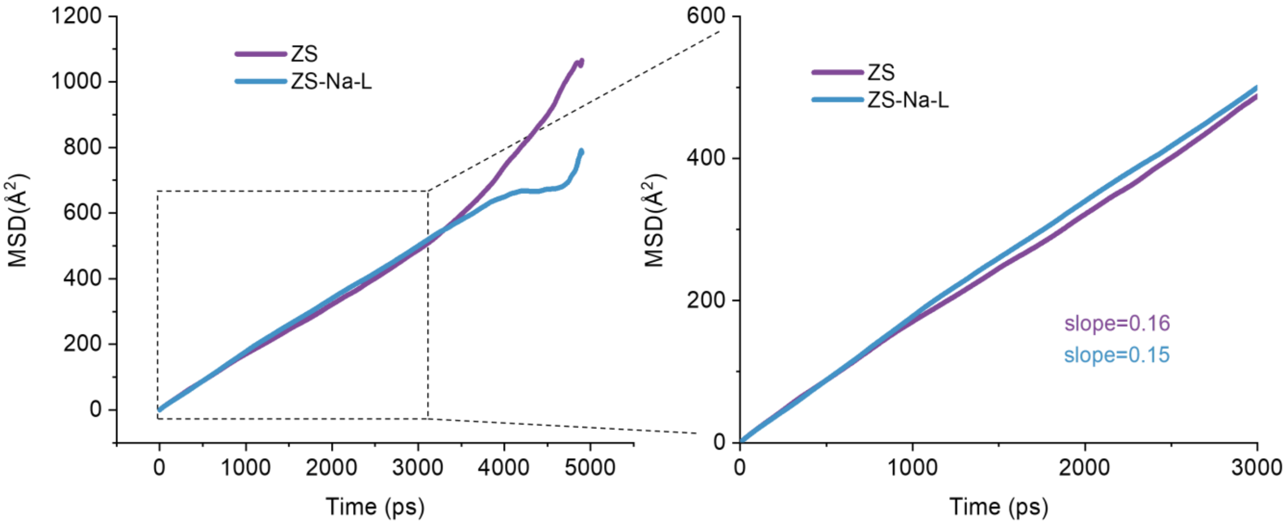

Fig. S14 MSD as a function of time under ZS and ZS-Na-L electrolyte

The diffusion coefficient (D) can be calculated by equation:

$$D=\lim_{\Delta t\to\infty}\frac{MSD(\Delta t)}{2d\Delta t} \tag{S1}$$

where d is the space dimension, Hence the D of Zn^{2+} in ZS and ZS-Na-L is 2.5×10^{-10} and 2.7×10^{-10} m^2 s^{-1} respectively.

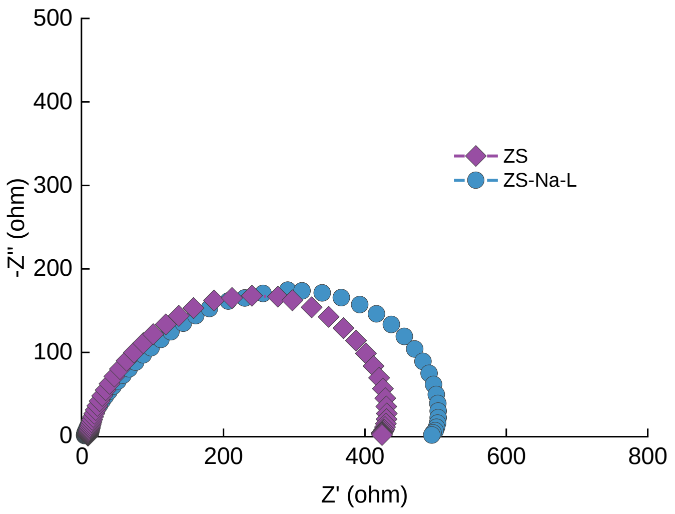

Fig. S15 EIS of Zn-Zn cells using ZS and ZS-Na-L electrolytes

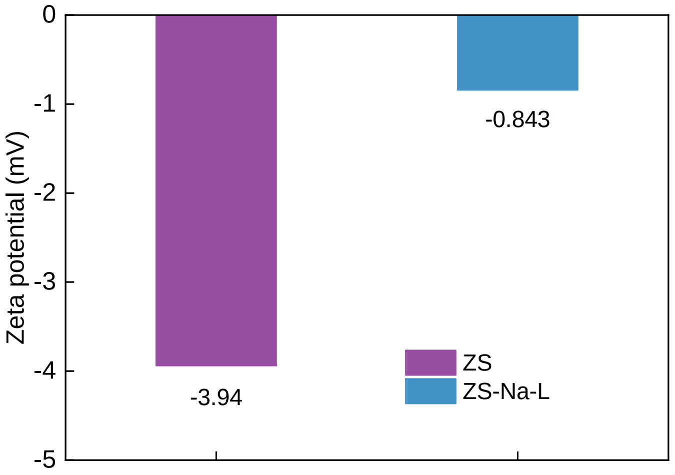

Fig. S16 The statistical Zeta potentials of Zn depositions (we disassembled the Zn||Ti cell after discharging 1 h at the current density of 2 mA cm^{-2}, took out the Ti electrode, and collected the deposited Zn metal on the Ti electrode) in ZS and ZS-Na-L electrolytes

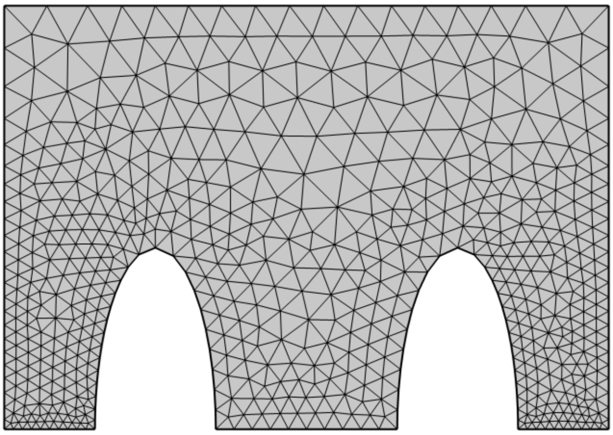

Fig. S17 Mesh distribution for finite element method simulations

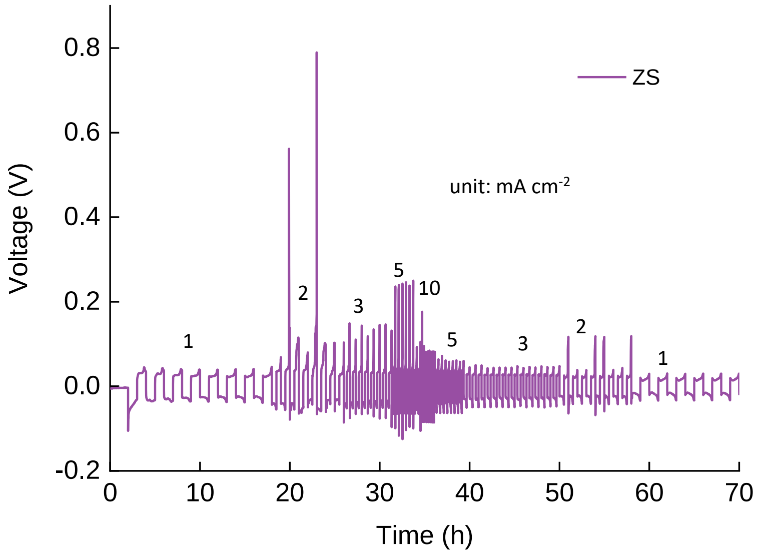

Fig. S18 Rate perfromance of Zn-Zn cells in ZS electrolyte

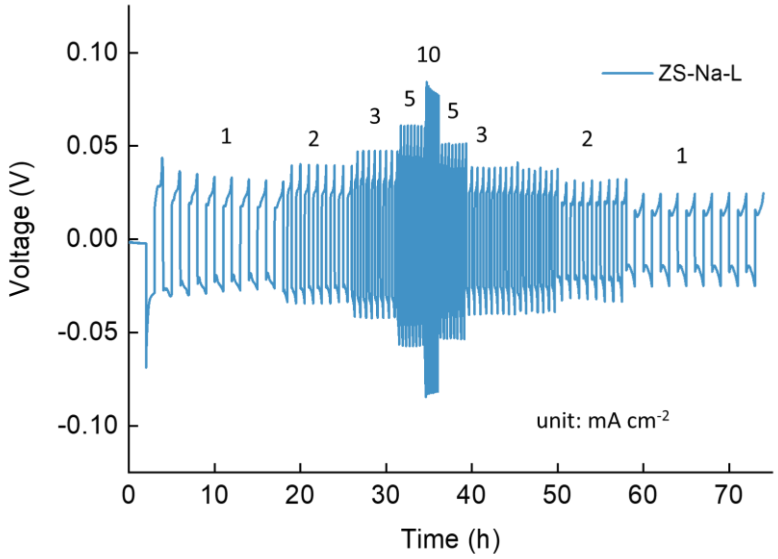

Fig. S19 Rate perfromance of Zn-Zn cells in ZS electrolyte

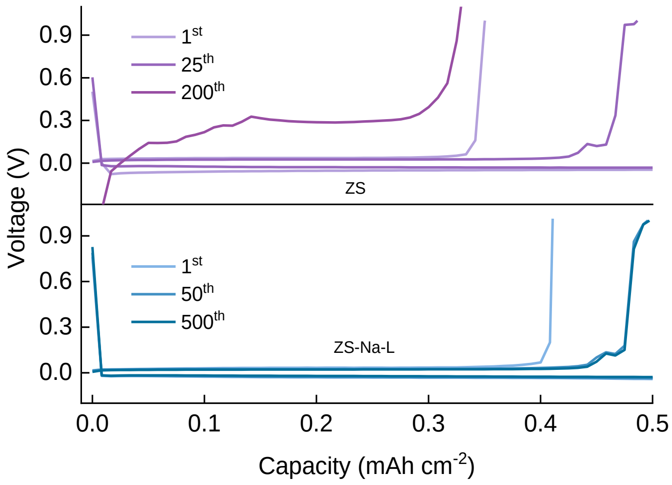

Fig. S20 voltage profiles at various cycles in Zn-Cu cells

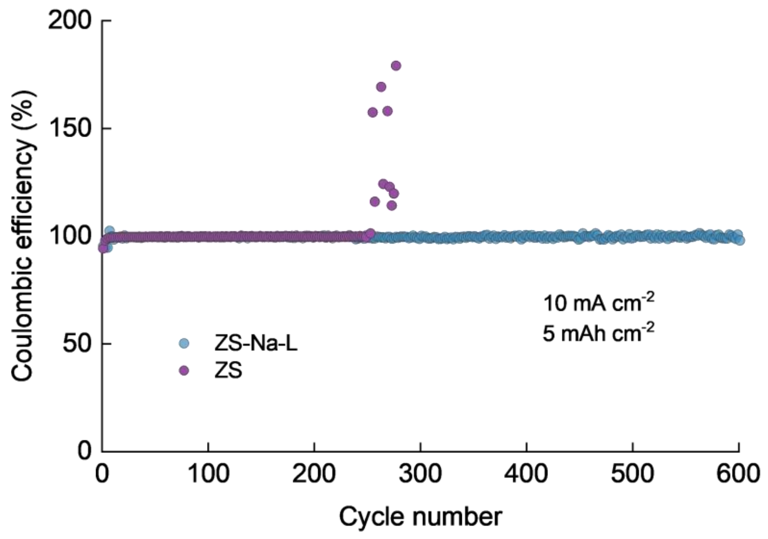

Fig. S21 Zn plating/stripping CE at 10 mA cm^{-2} and 5 mAh cm^{-2} in different electrolytes

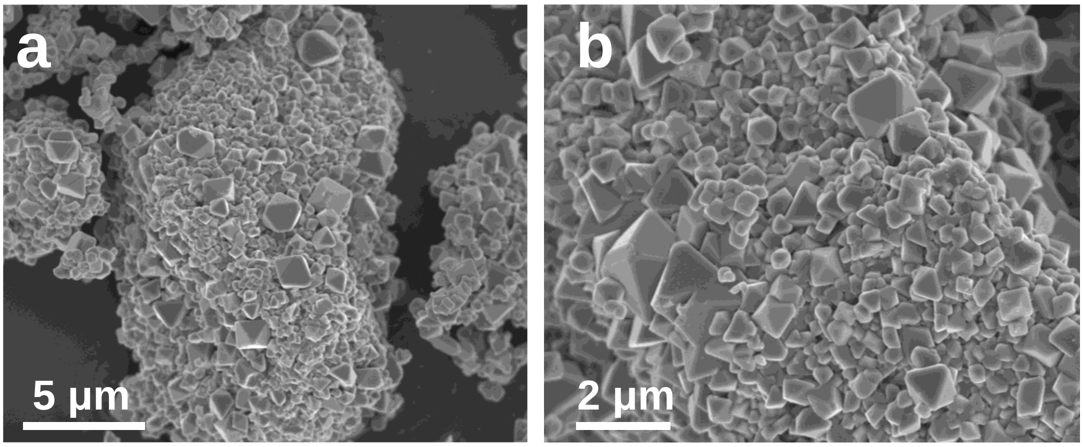

Fig. S22 SEM image of commercial LMO powders

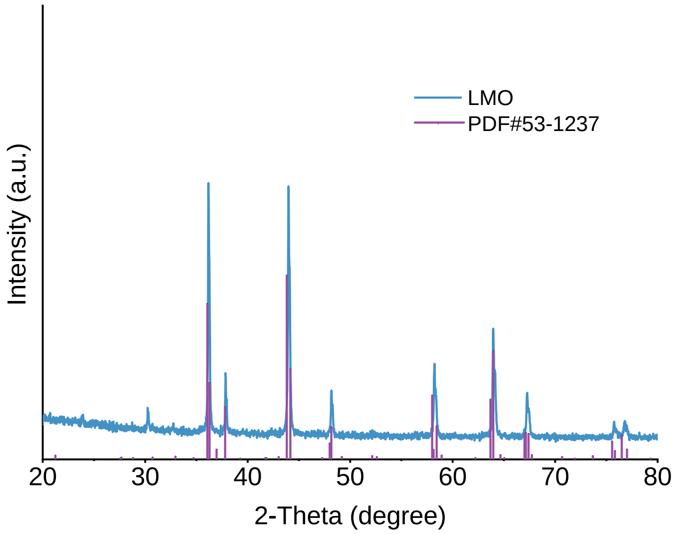

Fig. S23 XRD pattern of commercial LMO powders

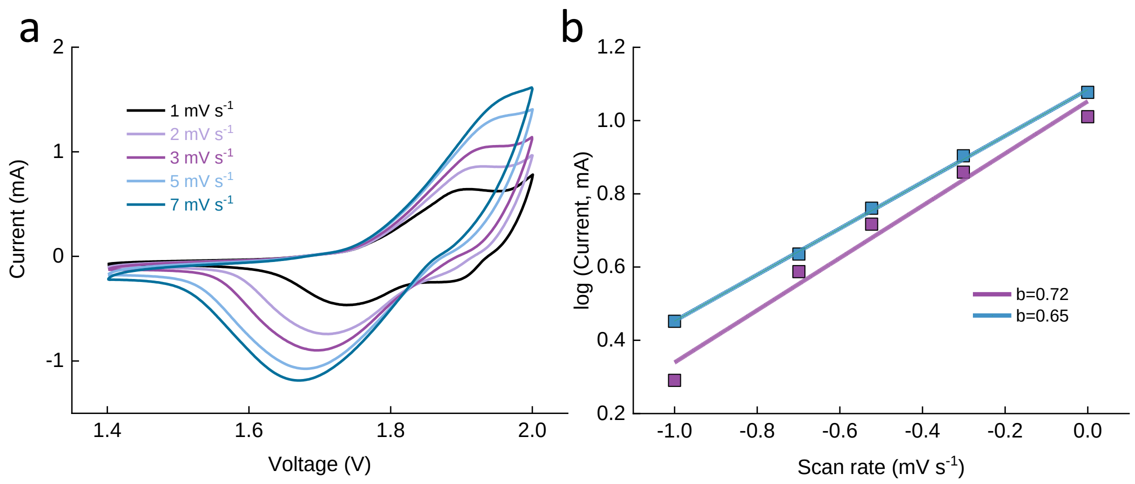

Fig. S24 **a** CV curves of Zn-LMO cells in ZS-Na-L electrolyte; **b** log(i)-log(v) plots at corresponded peak currents of Zn-LMO cells

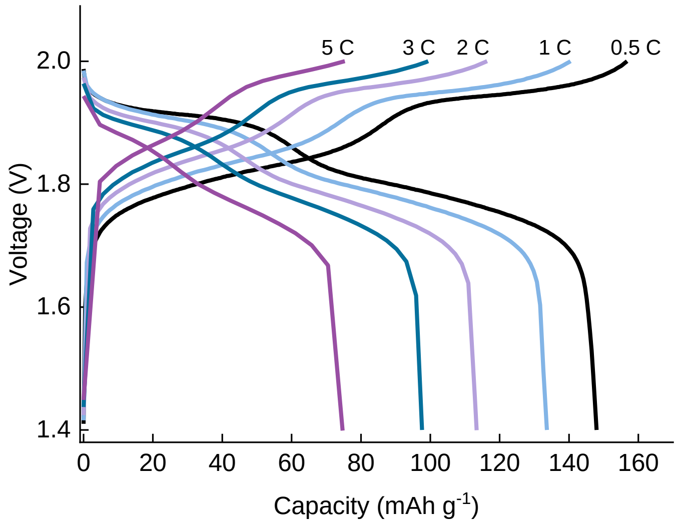

Fig. S25 GCD curves of Zn-LMO cells based on ZS-Na-L electrolyte at various current densities

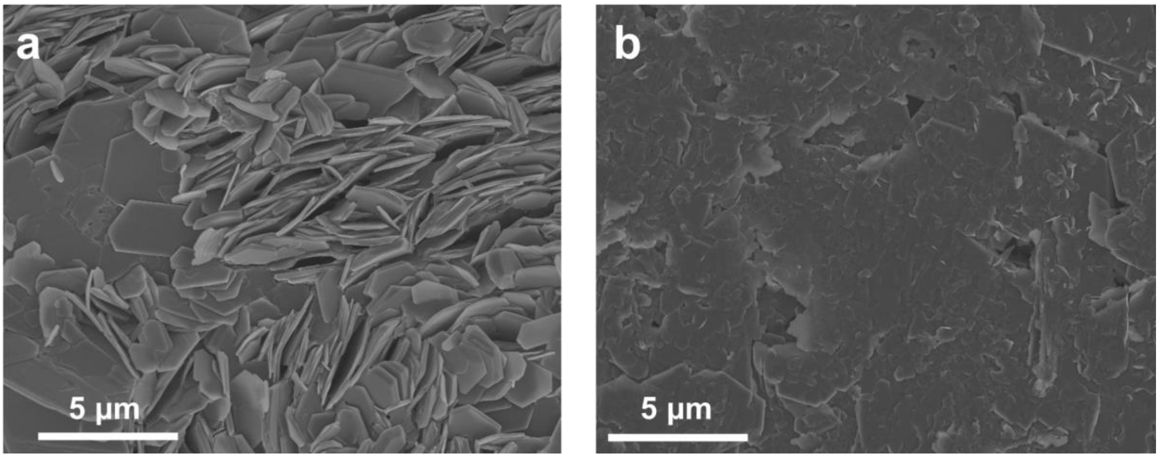

Fig. S26 SEM images of Zn electrode in full cells after cycling using **a** ZS electrolyte and **b** ZS-Na-L electrolyte

Table S1 Fitting results for symmetric cells at different temperatures

Symmetrical cells	Res	20 °C	30 °C	40 °C	50 °C
ZS electrolyte	R_{ct}	812.4	445.8	270.4	148.4
ZS-Na-L electrolyte	R_{ct}	982.8	544.5	330.2	164.2

Table S2 CPC comparison of Zn-Zn cell in recent reports

**No.**	**Modified strategy**	**Current density: mA cm^{-2}**	**Capacity: Ah cm^{-2}**	**Refs.**
This work	Na-L additive	10	2.5	-
This work	Na-L additive	20	2	-
1	Cyclodextrins additives	10	0.5	[S1]
2	polyacrylamide additive	20	1	[S2]
3	saturated fatty acid-zinc interphases	1	1	[S3]
4	sulfonate anion texturing	1	0.4	[S4]
5	$Ti_3C_2T_x$ MXene additive	2	1.2	[S5]
6	Glucose additive	5	0.687	[S6]
7	tripropylene glycol additive	2	1	[S7]

Supplementary References

[S1] K. Zhao, G. Fan, J. Liu, F. Liu, J. Li et al., Boosting the kinetics and stability of zn anodes in aqueous electrolytes with supramolecular cyclodextrin additives. J. Am. Chem. Soc. **144**, 11129-11137 (2022). http://doi.org/10.1021/jacs.2c00551

[S2] Q. Zhang, J. Luan, L. Fu, S. Wu, Y. Tang et al., The three-dimensional dendrite-free zinc anode on a copper mesh with a zinc-oriented polyacrylamide electrolyte additive. Angew. Chem. Int. Ed. **58**, 15841-15847 (2019). http://doi.org/10.1002/anie.201907830

[S3] M. Fu, H. Yu, S. Huang, Q. Li, B. Qu et al., Building sustainable saturated fatty acid-zinc interfacial layer toward ultra-stable zinc metal anodes. Nano Lett. **23**, 3573-3581 (2023). http://doi.org/10.1021/acs.nanolett.3c00741

[S4] D. Yuan, J. Zhao, H. Ren, Y. Chen, R. Chua et al., Anion texturing towards dendrite-free zn anode for aqueous rechargeable batteries. Angew. Chem. Int. Ed. **60**, 7213-7219 (2021). http://doi.org/10.1002/anie.202015488

[S5] C. Sun, C. Wu, X. Gu, C. Wang, Q. Wang, Interface engineering via $ti_3c_2t_x$ mxene electrolyte additive toward dendrite-free zinc deposition. Nano-Micro Lett. **13**, 89 (2021). http://doi.org/10.1007/s40820-021-00612-8

[S6] P. Sun, L. Ma, W. Zhou, M. Qiu, Z. Wang et al., Simultaneous regulation on solvation shell and electrode interface for dendrite-free zn ion batteries achieved by a low-cost glucose additive. Angew. Chem. Int. Ed. **60**, 18247-18255 (2021). http://doi.org/10.1002/anie.202105756

[S7] Z. Liu, R. Wang, Q. Ma, J. Wan, S. Zhang et al., A dual-functional organic electrolyte additive with regulating suitable overpotential for building highly reversible aqueous zinc ion batteries. Adv. Funct. Mater. 2214538 (2023). http://doi.org/10.1002/adfm.202214538
